# Supplementary material for: Independent somatic evolution underlies clustered neuroendocrine tumors in the human small intestine
Source: Nat Commun. 2021 Nov 4;12:6367. doi: 10.1038/s41467-021-26581-5 (PMC8568927; doi:10.1038/s41467-021-26581-5)
Supplement: Supplementary file 1 — Supplementary Information [file 41467_2021_26581_MOESM1_ESM.pdf]

Elias, Ardalan et al.

Supplementary appendix

Table of Contents

Supplementary Figure 1 2
Supplementary Figure 2 3
Supplementary Figure 3 4
Supplementary Figure 4 5
Supplementary Figure 5 6
Supplementary Figure 6 7
Supplementary Figure 7 8
Supplementary Figure 8 9
Supplementary Figure 9 10
Supplementary Figure 10 11
Supplementary Figure 11 12
Supplementary Figure 12 13
Supplementary Figure 13 14
Supplementary Figure 14 15
Supplementary Figure 15 16
Supplementary Figure 16 17
Supplementary Figure 17 18
Supplementary Figure 18 19
Supplementary Figure 19 20
Supplementary Table 1 21

Supplementary Figure 1

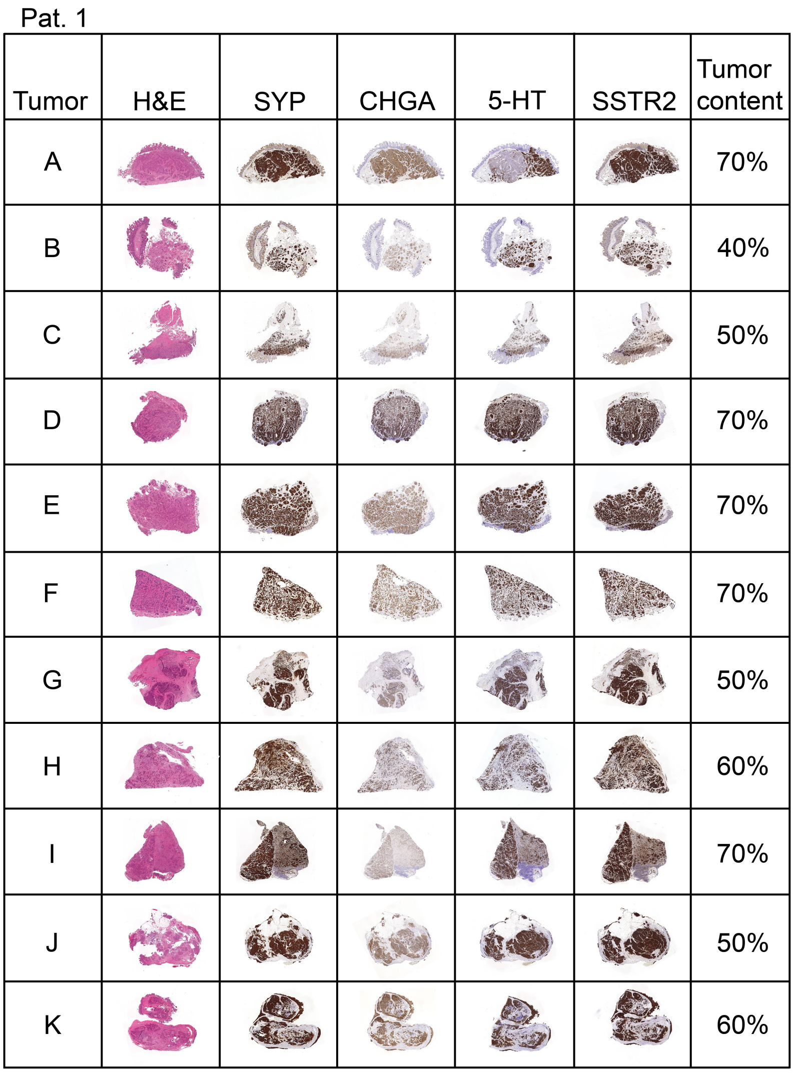

Immunohistochemical analysis of all sequenced samples from Patient 1. All tumors display similar morphology, and all express the neuroendocrine markers, SYP (synaptophysin), CHGA (chromogranin A) and 5-HT (serotonin) as well as the clinically relevant SSTR2 (somatostatin receptor 2). The assessment of tumor cell content was performed visually on hematoxylin and eosin-stained (H&E) sections of the tumors. The letter in each row indicates the sample ID.

Supplementary Figure 2

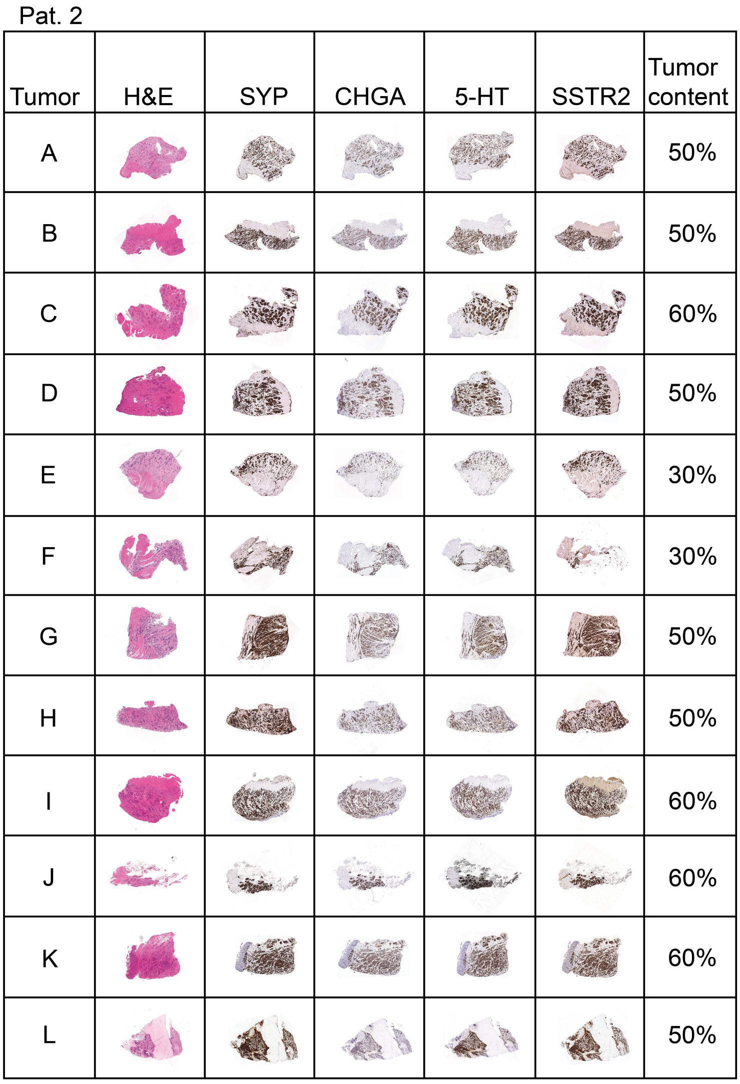

Immunohistochemical analysis of all sequenced samples from Patient 2. All tumors display similar morphology, and all express the neuroendocrine markers, SYP (synaptophysin), CHGA (chromogranin A) and 5-HT (serotonin) as well as the clinically relevant SSTR2 (somatostatin receptor 2). The assessment of tumor cell content was performed visually on hematoxylin and eosin-stained (H&E) sections of the tumors. The letter in each row indicates the sample ID.

Supplementary Figure 3

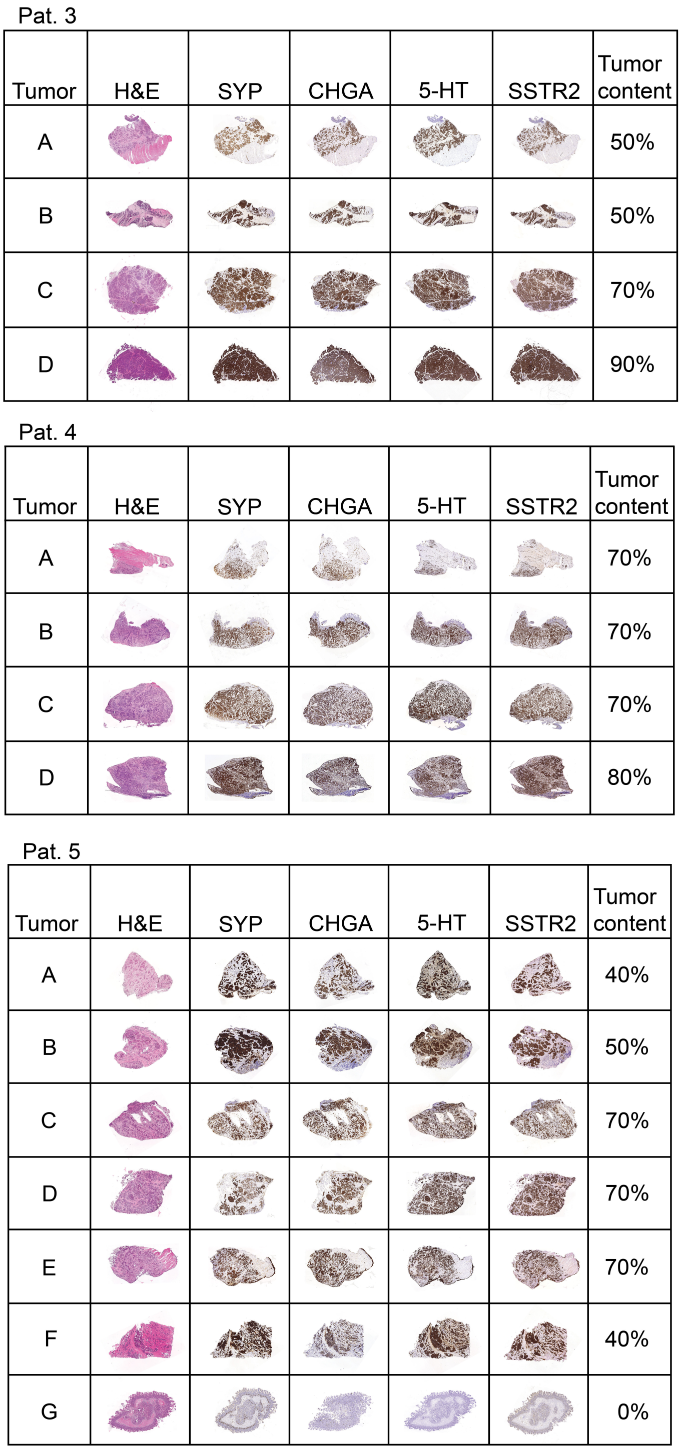

Immunohistochemical analysis of all sequenced samples from Patient 3-5. All tumors display similar morphology, and all express the neuroendocrine markers, SYP (synaptophysin), CHGA (chromogranin A) and 5-HT (serotonin) as well as the clinically relevant SSTR2 (somatostatin receptor 2). The assessment of tumor cell content was performed visually on hematoxylin and eosin-stained (H&E) sections of the tumors. The letter in each row indicates the sample ID. Sample 5G is from normal intestinal mucosa.

Supplementary Figure 4

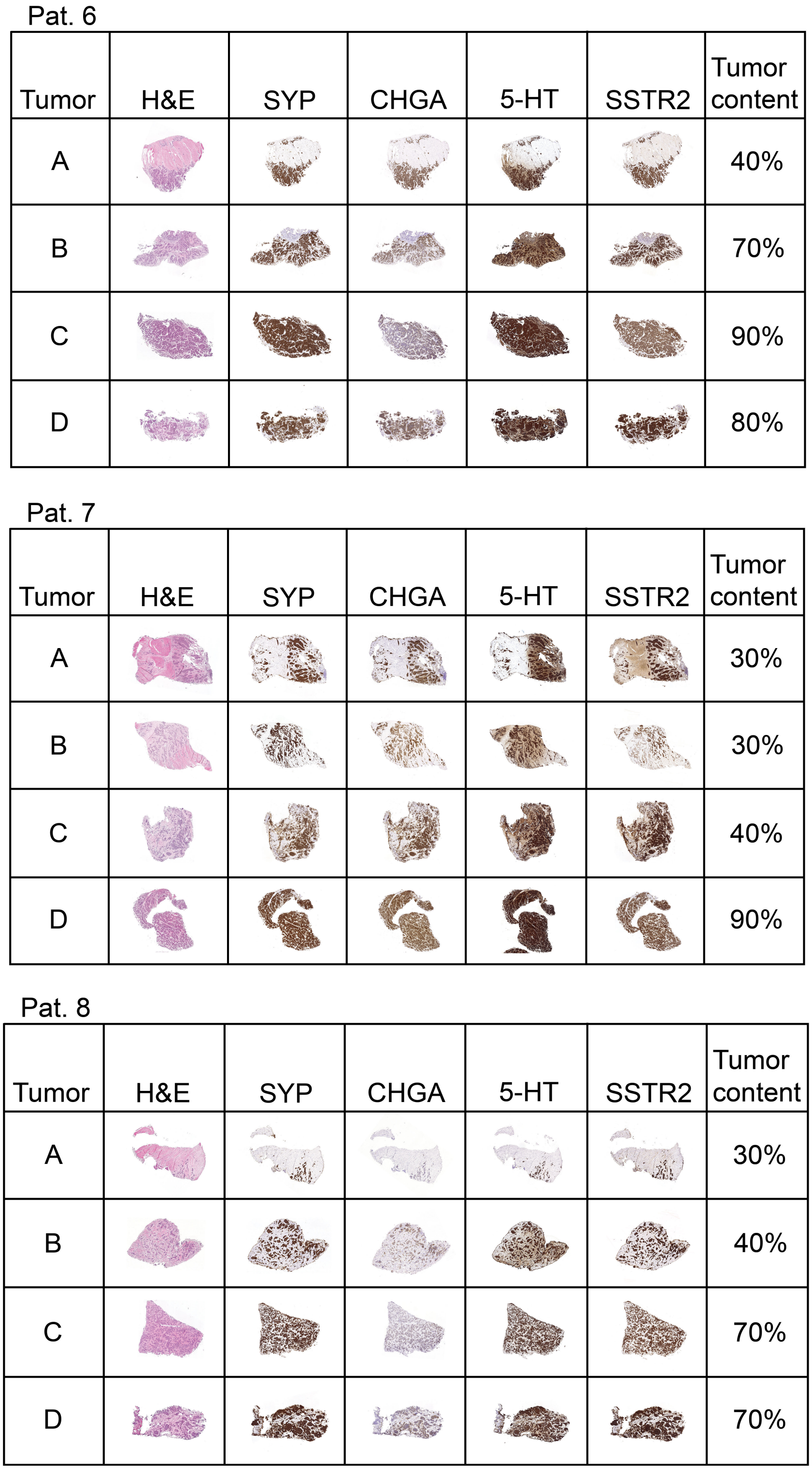

Immunohistochemical analysis of all sequenced samples from Patient 6-8. All tumors display similar morphology, and all express the neuroendocrine markers, SYP (synaptophysin), CHGA (chromogranin A) and 5-HT (serotonin) as well as the clinically relevant SSTR2 (somatostatin receptor 2). The assessment of tumor cell content was performed visually on hematoxylin and eosin-stained (H&E) sections of the tumors. The letter in each row indicates the sample ID.

Supplementary Figure 5

Pat. 9

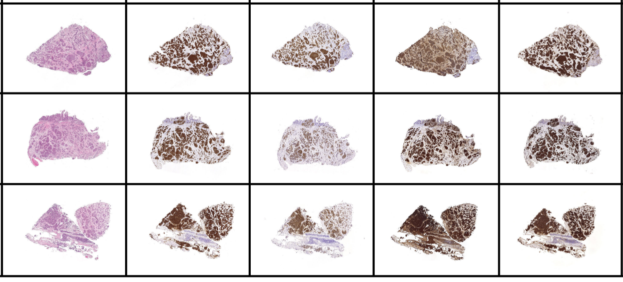

Tumor	H&E	SYP	CHGA	5-HT	SSTR2	Tumor content
A						70%
B						70%
C						70%

Pat. 10

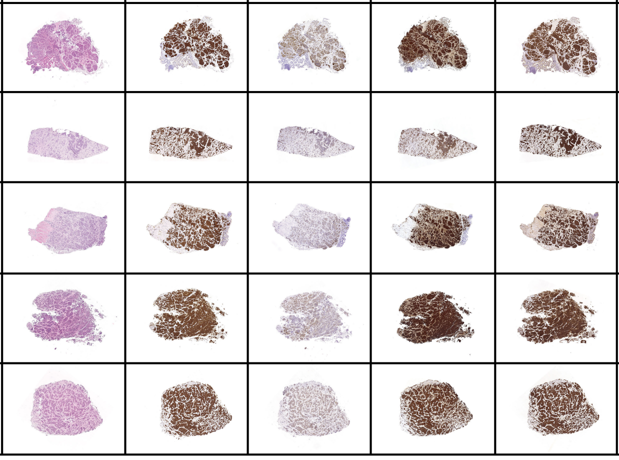

Tumor	H&E	SYP	CHGA	5-HT	SSTR2	Tumor content
A						80%
B						40%
C						50%
D						90%
E						80%

Pat. 11

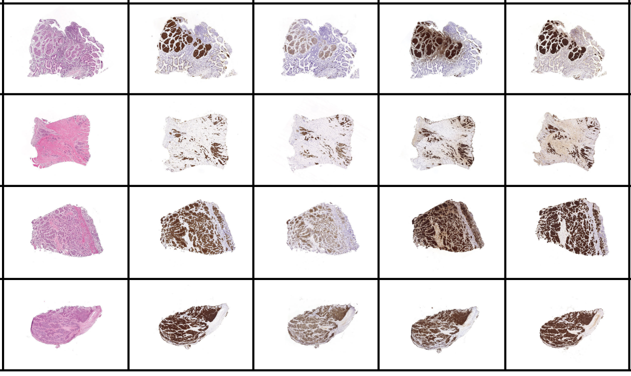

Tumor	H&E	SYP	CHGA	5-HT	SSTR2	Tumor content
A						50%
B						30%
C						80%
D						80%

Immunohistochemical analysis of all sequenced samples from Patient 9-11. All tumors display similar morphology, and all express the neuroendocrine markers, SYP (synaptophysin), CHGA (chromogranin A) and 5-HT (serotonin) as well as the clinically relevant SSTR2 (somatostatin receptor 2). The assessment of tumor cell content was performed visually on hematoxylin and eosin-stained (H&E) sections of the tumors. The letter in each row indicates the sample ID.

Supplementary Figure 6

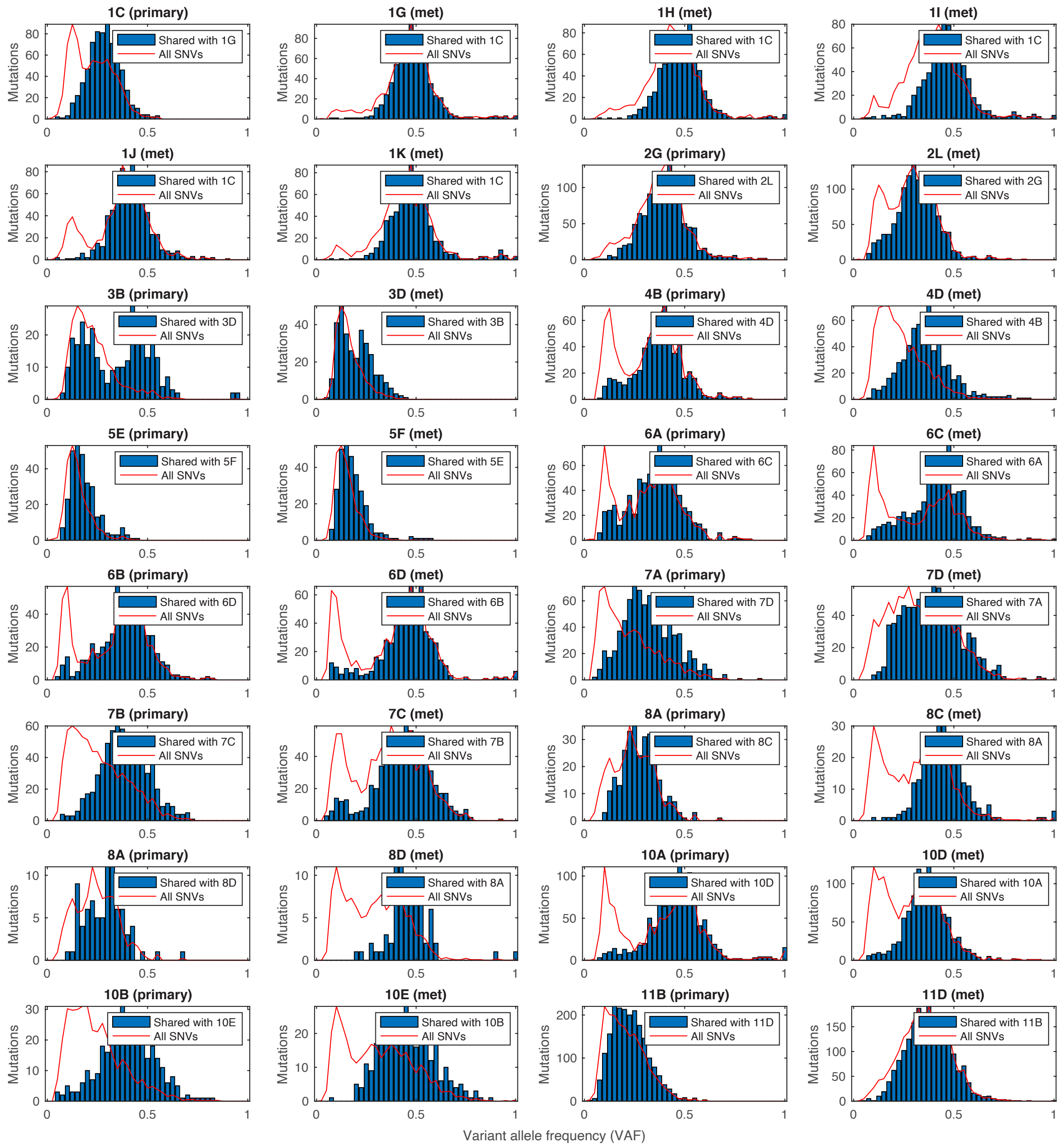

Analysis of variant allele frequency (VAF) distributions of primary-metastasis shared SNVs. VAF distribution plots of SNVs shared between primary tumors and corresponding metastases in all relevant samples. The overall VAF distribution for all high-confidence mutations in each sample is shown as reference (red; normalized to fit y-axis scale). More detailed views and discussions of Patient 3 and Patient 7 are provided in **Supplementary Figs. 14 and 15**. Source data are provided as a Source Data file.

Supplementary Figure 7

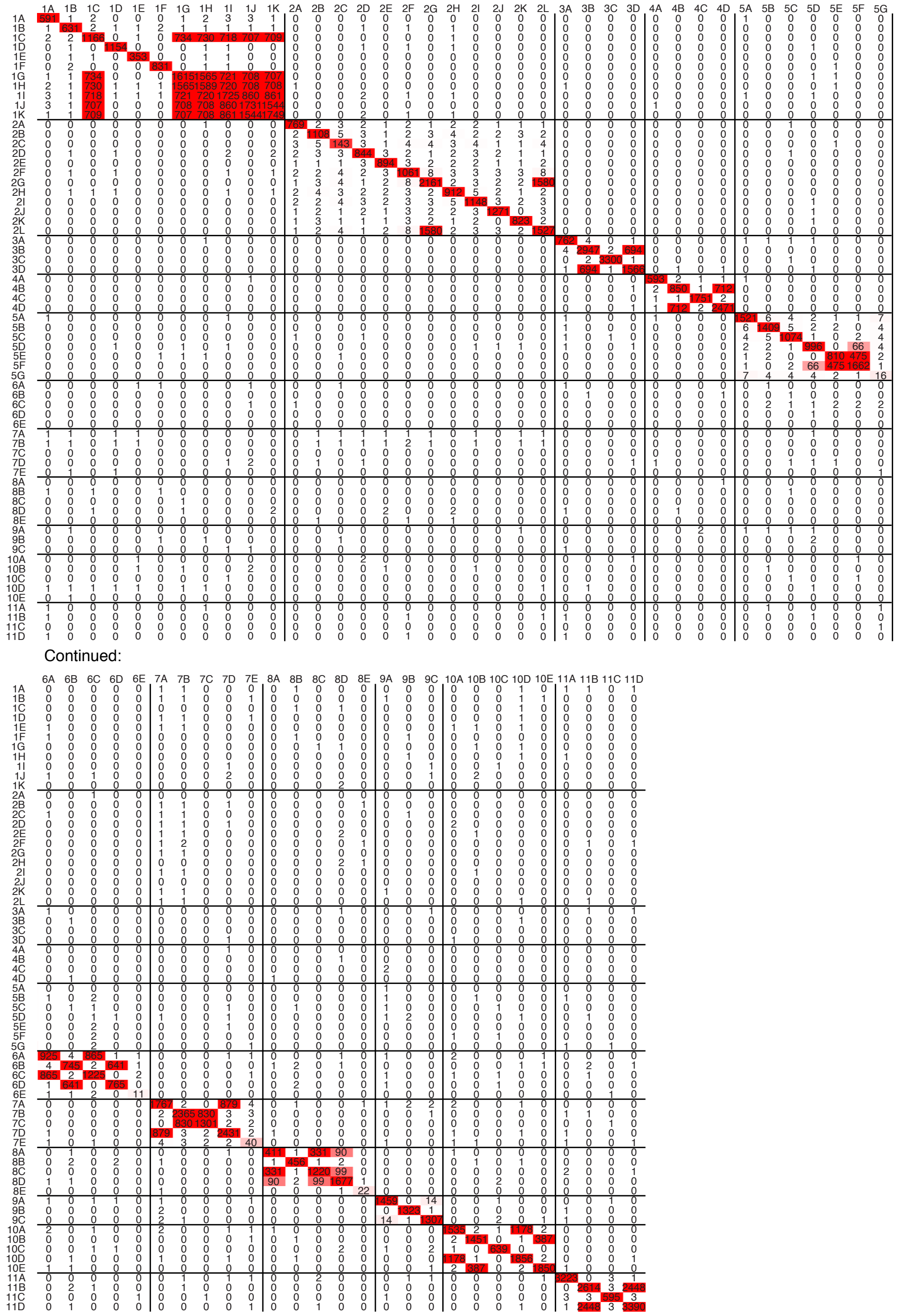

High-sensitivity search for shared variants by whitelisting of SNVs called at high confidence in at least one sample. A two-step procedure was used, where any somatic SNVs called at high confidence (detected by both Mutect and VarScan using various filters including a strand filter as described in Methods) in at least one sample were “whitelisted”. This was followed by a more sensitive search for presence of the whitelisted variants using VarScan without stringent filters (e.g. exclusion of strand filter, see Methods). The number and letter in each row/column label indicates the patient number and sample ID, respectively. Source data are provided as a Source Data file.

Supplementary Figure 8

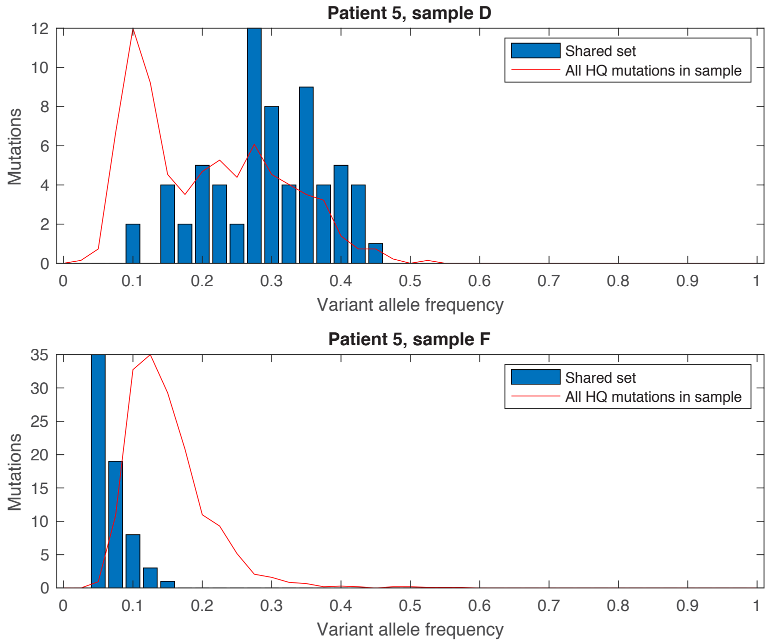

Variant allele frequency (VAF) distributions indicate low-level contamination between samples D and F from Patient 5. Samples D (primary tumor) and F (lymph node metastasis) in Patient 5 shared 8 SNVs in the main phylogenetic analysis (main **Fig. 2**). A high-sensitivity search for shared variants (**Supplementary Fig. 7**) revealed 58 additional shared variants called at high confidence in at least one of the samples while being detectable using relaxed filters (e.g. exclusion of strand filter, see Methods) in the other sample. These 66 shared SNVs were present at high VAFs in sample D (top histogram) while being highly subclonal in sample F (bottom histogram). This supports that the metastasis sample (F) was contaminated with DNA from D, for example during sample handling or, alternatively, that trace amounts of material from D reached F through metastatic spread. Overall VAF distributions for all high-quality mutations in each sample is shown for comparison (red; normalized to fit y-axis scale). Source data are provided as a Source Data file.

Supplementary Figure 9

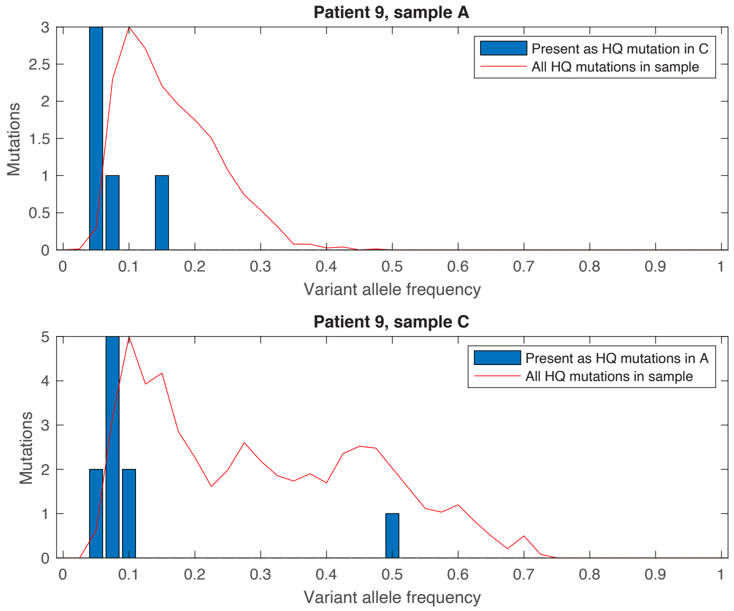

Variant allele frequency (VAF) distributions indicate low-level contamination between samples A and C from Patient 9. Samples A (primary tumor) and C (lymph node metastasis) in Patient 9 shared a single variant in the main phylogenetic analysis (main **Fig. 2**). A high-sensitivity search for subclonal shared SNVs (**Supplementary Fig. 7**) revealed 13 additional variants shared between these samples, all present at high confidence in at least one of the samples while being detectable using relaxed filters (e.g. exclusion of strand filter, see Methods) in the other. The graphs show that variants present at high confidence in A was generally found at low VAF in C, and vice versa. A likely explanation is sample contamination, although transfer of trace amounts of tumor material through metastatic spread could in principle also explain these patterns. Overall VAF distributions for all high-quality mutations in each sample is shown for comparison (red; normalized to fit y-axis scale). Source data are provided as a Source Data file.

Supplementary Figure 10

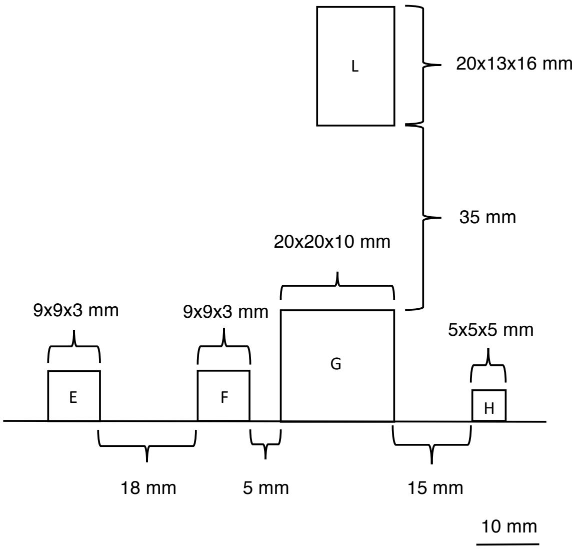

Physical positioning of tumors F, G and L in Patient 2.

Supplementary Figure 11

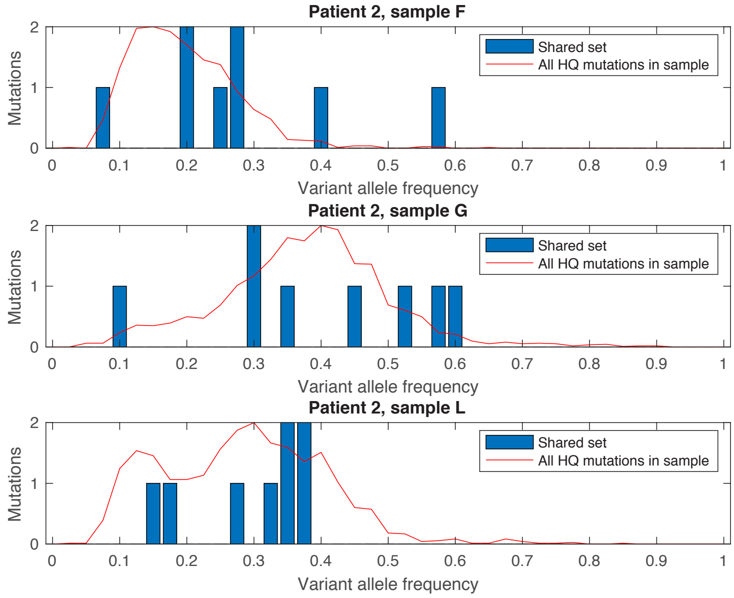

Variant allele frequency (VAF) distributions for 8 variants found in samples F, G and L from Patient 2. A set of 8 SNVs was shared between samples F, G and L (lymph node metastasis) in Patient 2 (main **Fig. 2**). This number stayed constant when using a high-sensitivity approach to uncover subclonal shared SNVs (**Supplementary Fig. 7**). Manual inspection in IGV supported that the shared calls were mostly of high quality, with only one having characteristics of being a false positive call (**Supplementary Data 2**). The shared SNVs showed normal VAF distributions (comparable to other mutations) in all samples. The overall VAF distribution for all high-quality mutations in each sample is shown for comparison (red; normalized to fit y-axis scale). Source data are provided as a Source Data file.

Supplementary Figure 12

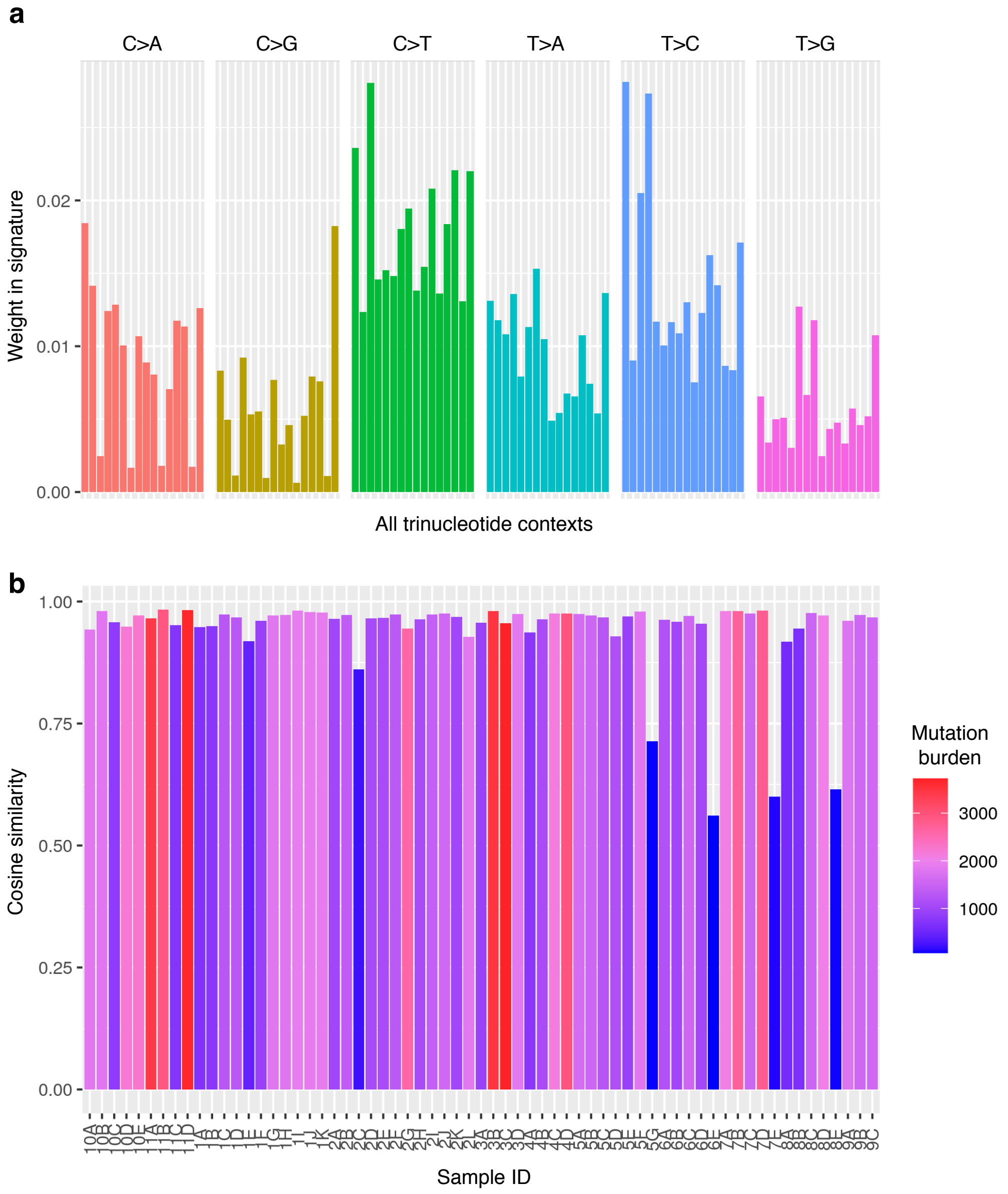

Highly coherent trinucleotide substitution profile across the cohort. (**a**) Average trinucleotide substitution profile in the cohort. (**b**) Cosine similarity of per-sample trinucleotide profiles and the cohort average shown in (a). Most samples showed high similarity, and deviation from the average profile was associated with reduced mutation burden, thus reducing the ability to accurately determine the signature. This is most clearly seen for the normal mucosa tissue samples, which all have low mutation counts. Source data are provided as a Source Data file.

Supplementary Figure 13

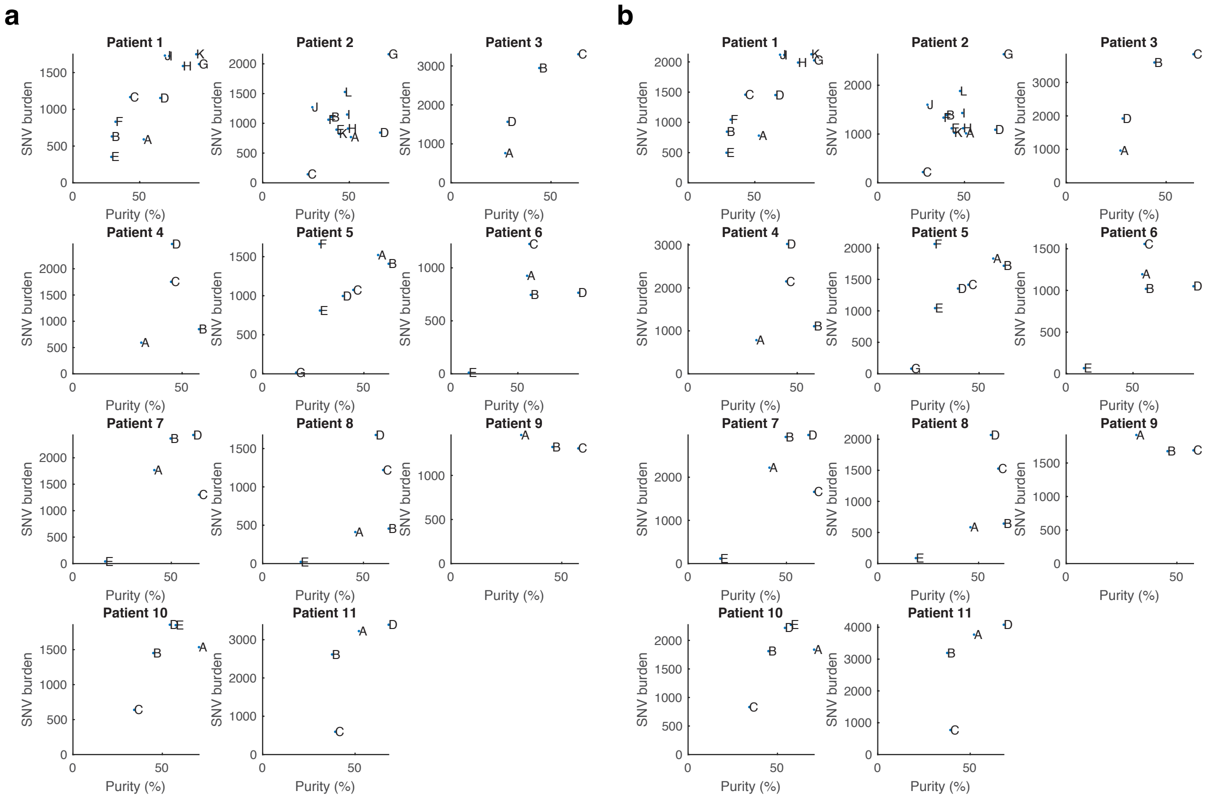

Positive correlations between per-sample estimated purity and average variant allele frequency (VAF). Tumor purity, as determined by PurBayes, generally showed positive correlation with SNV burden. (**a**) Results using SNV calls used for phylogenetic analyses, which were stringently filtered for known population variants (dbSNP150 and ENSEMBL variants). (**b**) Results using relaxed population variant filtering (dbSNP138), as used for driver mutations and mutational signature analyses, were nearly identical. Source data are provided as a Source Data file.

Supplementary Figure 14

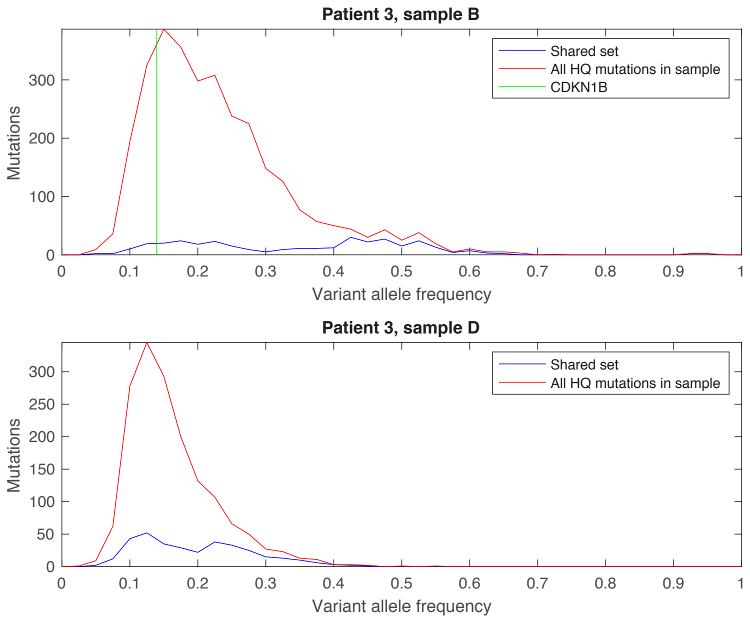

Variant allele frequency (VAF)-based analysis of a *CDKN1B* mutation in Patient 3. A *CDKN1B* SNV present in the metastatic primary tumor B is one of a large number of private variants (not shared with the metastasis D) present at relatively low VAF compared to shared variants, which showed higher mean VAF and bimodality in the primary tumor B. This may indicate subclonality, early whole genome duplication, or a combination of both. Source data are provided as a Source Data file.

Supplementary Figure 15

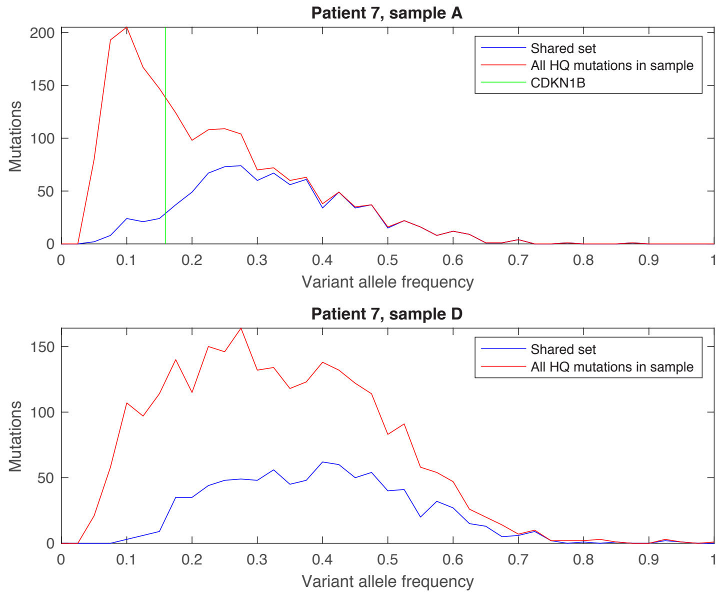

Variant allele frequency (VAF)-based analysis of a *CDKN1B* mutation in Patient 7. A *CDKN1B* SNV present in the metastatic primary tumor A is one of a many private variants (not shared with the metastasis D) present at relatively low VAF compared to shared variants. Source data are provided as a Source Data file.

Supplementary Figure 16

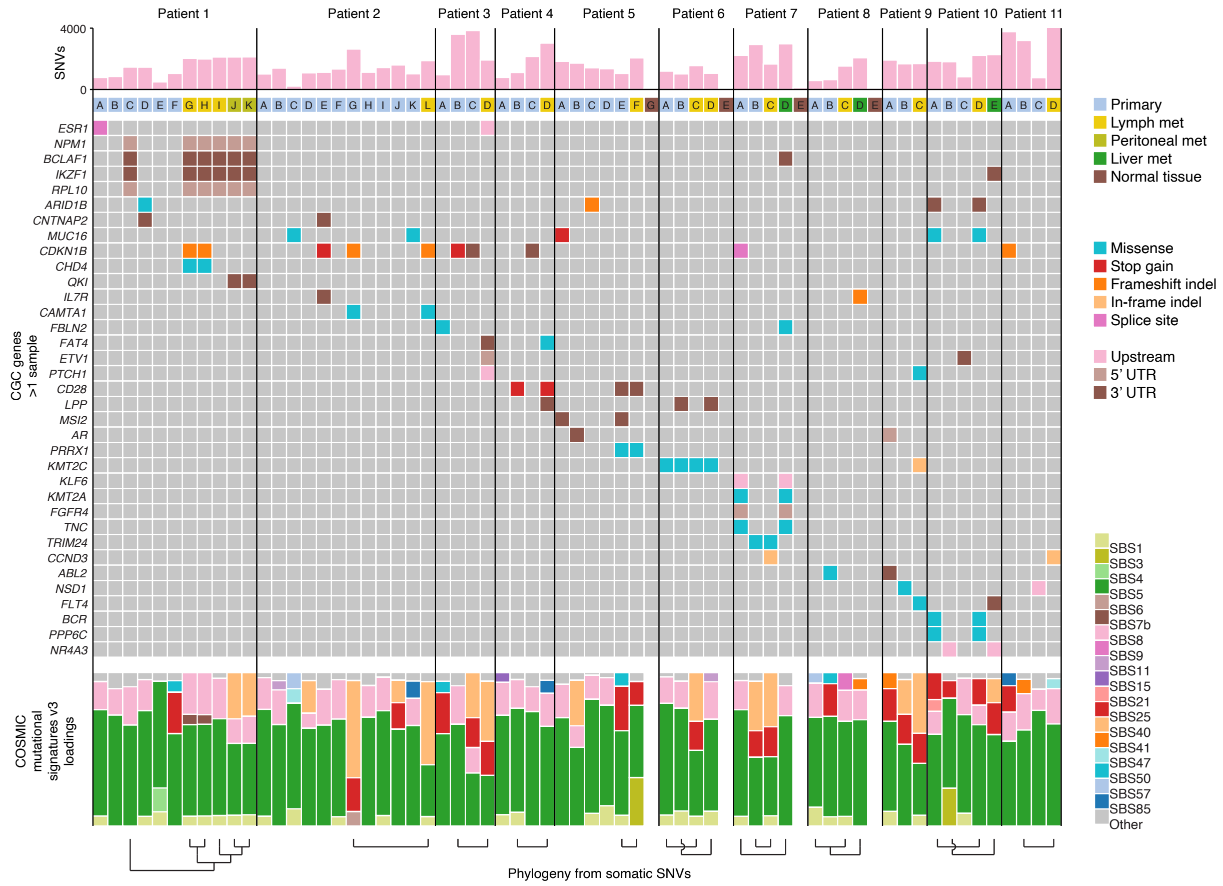

Overview of potential driver mutations including regulatory (upstream and UTR) mutations for all 11 patients. All CGC genes with non-synonymous mutations in more than one sample are shown. Upstream and UTR mutations are shown as reported by AnnoVar. Phylogenetic relationships previously inferred from somatic SNVs (main **Fig. 1-2**) are indicated at the bottom. Somatic SNV burdens, potential driver mutations (Cancer Gene Census genes mutated in >1 sample) and mutational signature loadings in this figure were determined using less rigorous population variant filtering compared to the phylogenetic analyses (see Methods). CGC, Cancer Gene Census. Source data are provided as a Source Data file.

Supplementary Figure 17

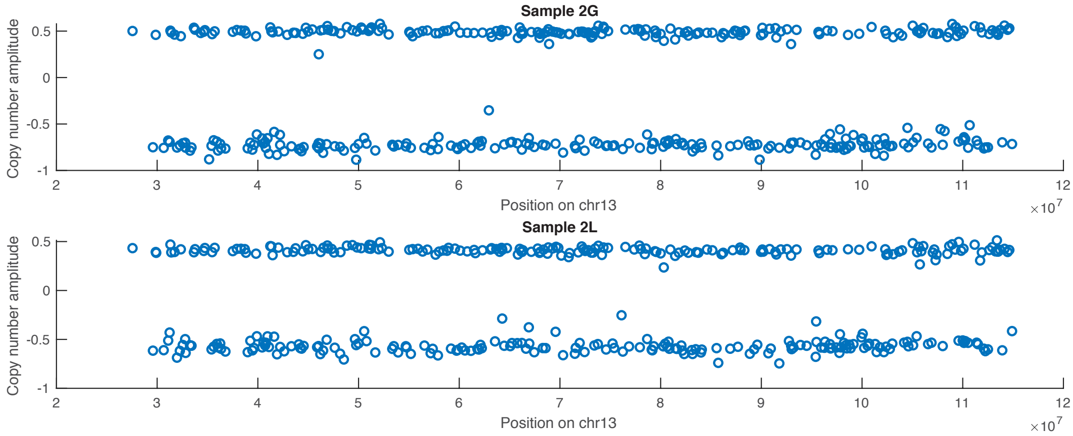

Clustered copy number alterations on chromosome 13 in Patient 2 compatible with chromothripsis. CNAs on chromosome 13 in Patient 2 (samples G and L) oscillated between two distinct amplitude states, which is a hallmark of chromothripsis. Source data are provided as a Source Data file.

Supplementary Figure 18

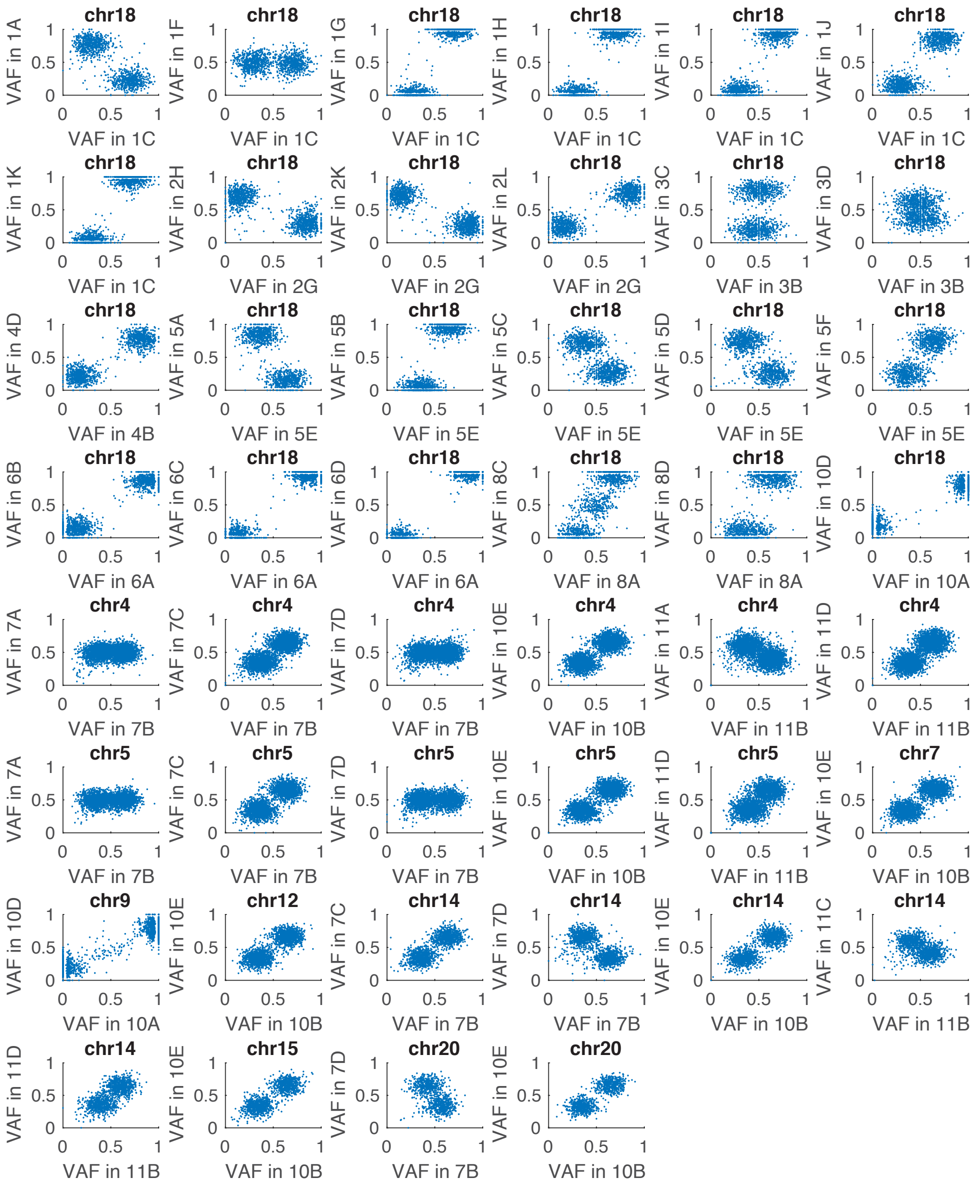

Chromosomal phasing of whole-chromosome copy number events based on germline heterozygous SNPs. Heterozygous germline SNPs on chr18 (read coverage 20 and VAF ranging from 0.25 to 0.75 in the blood normal) were used to determine what chromosome homolog was altered. The scatter plots compare VAFs for individual SNPs in a reference sample compared to other relevant samples. The data was down-sampled to 2% to reduce dot density in the plots. The related samples 7A and 7D lacked VAF bimodality on chr4/5 indicative of biallelic gain, further supported by high copy number amplitude in these samples. Lack of bimodality on chr18 in the related samples 3B and 3D may be explained by a biallelic deletion on a whole genome duplication background (notably the unrelated sample, 3C, deviates and shows bimodality). Alternative explanations include a homozygous deletion (unlikely due to low-level amplitude change in 3D) or a false copy number call (unlikely due to strong amplitude change in 3B). Source data are provided as a Source Data file.

Supplementary Figure 19

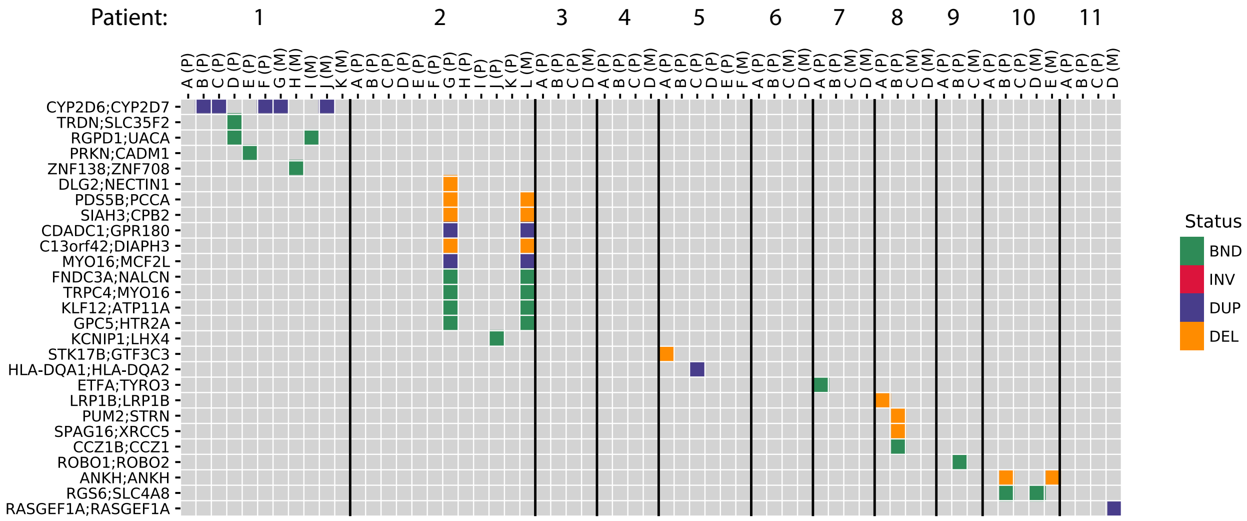

Analysis of somatic genomic structural alterations. In-frame fusion gene pairs colored by alteration type, with translocations (BND) shown in green, inversions in red, duplications in purple, and deletions in orange. Source data are provided as a Source Data file.

Supplementary Table 1

Patient characteristics summary

**Age at the time of surgery (years)**	Median (range)	76 (48-81)
**Gender**	Female (%)<br>Male (%)	6 (55%)<br>5 (45%)
**Family history of SI-NET or other endocrine tumors**	Yes / No	0 / 11
**Radical surgery (R0)**<br>**Grade (WHO 2010)**	Yes / No<br>Median (range)	7 / 4<br>1 (1-2)
