# Supplementary material for: Independent somatic evolution underlies clustered neuroendocrine tumors in the human small intestine
Source: Nat Commun. 2021 Nov 4;12:6367. doi: 10.1038/s41467-021-26581-5 (PMC8568927; doi:10.1038/s41467-021-26581-5)
Supplement: Supplementary file 3 — Description of Additional Supplementary Files [file 41467_2021_26581_MOESM3_ESM.pdf]

Description of Additional Supplementary Files

File Name: Supplementary Data 1
Description: Sample overview and WGS statistics

File Name: Supplementary Data 2
Description: Manual inspection of 29 remaining spurious overlapping SNVs (appearing in pairs sharing <10 mutations) in high-confidence mutation calls

File Name: Supplementary Data 3
Description: Somatic non-synonymous, splicing, upstream (promoter) or UTR mutations by gene based on calls filtered for dbSNP138 population variants
