# Supplementary material for: Independent somatic evolution underlies clustered neuroendocrine tumors in the human small intestine
Source: Nat Commun. 2021 Nov 4;12:6367. doi: 10.1038/s41467-021-26581-5 (PMC8568927; doi:10.1038/s41467-021-26581-5)
Supplement: Supplementary file 5 — Reporting Summary [file 41467_2021_26581_MOESM5_ESM.pdf]

nature portfolio

Corresponding author(s): Erik Larsson

Last updated by author(s): Sep 15, 2021

Reporting Summary

Nature Portfolio wishes to improve the reproducibility of the work that we publish. This form provides structure for consistency and transparency in reporting. For further information on Nature Portfolio policies, see our Editorial Policies and the Editorial Policy Checklist.

Statistics

For all statistical analyses, confirm that the following items are present in the figure legend, table legend, main text, or Methods section.

n/a	Confirmed	
☒	☐	The exact sample size (*n*) for each experimental group/condition, given as a discrete number and unit of measurement
☒	☐	A statement on whether measurements were taken from distinct samples or whether the same sample was measured repeatedly
☐	☒	The statistical test(s) used AND whether they are one- or two-sided<br>*Only common tests should be described solely by name; describe more complex techniques in the Methods section.*
☒	☐	A description of all covariates tested
☒	☐	A description of any assumptions or corrections, such as tests of normality and adjustment for multiple comparisons
☒	☐	A full description of the statistical parameters including central tendency (e.g. means) or other basic estimates (e.g. regression coefficient) AND variation (e.g. standard deviation) or associated estimates of uncertainty (e.g. confidence intervals)
☒	☐	For null hypothesis testing, the test statistic (e.g. *F*, *t*, *r*) with confidence intervals, effect sizes, degrees of freedom and *P* value noted<br>*Give P values as exact values whenever suitable.*
☒	☐	For Bayesian analysis, information on the choice of priors and Markov chain Monte Carlo settings
☒	☐	For hierarchical and complex designs, identification of the appropriate level for tests and full reporting of outcomes
☒	☐	Estimates of effect sizes (e.g. Cohen's *d*, Pearson's *r*), indicating how they were calculated

Our web collection on statistics for biologists contains articles on many of the points above.

Software and code

Policy information about availability of computer code

Data collection	None
Data analysis	Mutect2 (GATK v4.1.4.0), VarScan (v2.3.9), BWA as part of Sentieon Genomics Tools (bwa-mem v0.7.15.r1140 for patients 1-5 and 0.7.17.r1188 for patients 6-11), samtools (v1.9), ANNOVAR (v2019Oct24), PurBayes (v1.3), Computel (v1.2), TraFIC-mem, MEGAX (v10.1.8), deconstructSigs (v1.9.0), XCAVATOR (v2.1), Manta (v1.5.0), AnnotSV (v2.1), MATLAB (r2018a)

For manuscripts utilizing custom algorithms or software that are central to the research but not yet described in published literature, software must be made available to editors and reviewers. We strongly encourage code deposition in a community repository (e.g. GitHub). See the Nature Portfolio guidelines for submitting code & software for further information.

Data

Policy information about availability of data

All manuscripts must include a data availability statement. This statement should provide the following information, where applicable:

- Accession codes, unique identifiers, or web links for publicly available datasets
- A description of any restrictions on data availability
- For clinical datasets or third party data, please ensure that the statement adheres to our policy

The WGS sequencing data that support the findings in the article are deposited and available at the European Genome-Phenome Archive (EGA; https://ega-archive.org), which is hosted by the European Bioinformatics Institute (EBI) and the Centre for Genomic Regulation (CRG), through the primary accession code EGAS00001005096. Due to ethical and legal reasons, the data is deposited under controlled access. Data use conditions attached to this EGA dataset limits its use to approved users at a specific institution for a specific a health/medical/biomedical project and dictates that useful results should be made available to the wider scientific community. Access requests, which we aim to respond to within two weeks, should be addressed to Erik Elias (erik.elias@gu.se) or Erik Larsson

(erik.larsson@gu.se). The study makes use of SNP data from ENSEMBL/VEP (https://grch37.ensembl.org/Homo_sapiens/Tools/VEP; downloaded on Jan 13 2021) and dbSNP (https://www.ncbi.nlm.nih.gov/snp/; v150). Mutational signatures were downloaded from COSMIC (https://cancer.sanger.ac.uk/signatures/; v3 May 2019). Source data are provided with this paper.

Field-specific reporting

Please select the one below that is the best fit for your research. If you are not sure, read the appropriate sections before making your selection.

☒ Life sciences ☐ Behavioural & social sciences ☐ Ecological, evolutionary & environmental sciences

For a reference copy of the document with all sections, see nature.com/documents/nr-reporting-summary-flat.pdf

Life sciences study design

All studies must disclose on these points even when the disclosure is negative.

Sample size	11 patients (65 tumor or tissue samples) were analyzed in this study. This is an observational exploratory study reporting descriptive data. There is no attempt to establish causality of a predetermined exposure/intervention outcome and therefore there is no estimated effect size that enables sample size calculation. The study is presented as an initial case and a subsequent additional cohort reproducing the initial findings. The size of the subsequent cohort was ultimately arbitrarily determined and in part influenced by the availability of research material. Given the clear nature of the findings we believe that the cohort size is sufficient.
Data exclusions	No data were excluded.
Replication	A preliminary analysis was done based on one patient and 11 tumor samples, revealing that intestinal tumors were clonally independent. 10 additional patients (54 tumor or tissue samples) were then analyzed, and in all cases the initial finding was confirmed.
Randomization	This study did not attempt to associate causality with a specified intervention/exposure outcome and does not group the participants nor contain analysis of group outcomes. As a consequence, it is not possible to randomize participants in this study design.
Blinding	The study was not blinded. Some of the phylogenetics analyses were performed without knowledge of sample annotations; however, this was not documented or formalized in the study design prior to analysis, as is not typical for these types of exploratory studies.

Reporting for specific materials, systems and methods

We require information from authors about some types of materials, experimental systems and methods used in many studies. Here, indicate whether each material, system or method listed is relevant to your study. If you are not sure if a list item applies to your research, read the appropriate section before selecting a response.

Materials & experimental systems

n/a	Involved in the study	
☐	☒	Antibodies
☒	☐	Eukaryotic cell lines
☒	☐	Palaeontology and archaeology
☒	☐	Animals and other organisms
☐	☒	Human research participants
☒	☐	Clinical data
☒	☐	Dual use research of concern

Methods

n/a	Involved in the study	
☒	☐	ChIP-seq
☒	☐	Flow cytometry
☒	☐	MRI-based neuroimaging

Antibodies

Antibodies used	Anti-synaptophysin (SY38/M0776; Dako/Agilent)<br>Anti-chromogranin A (MAB319/PHE5; Chemicon)<br>Anti-5HT (H209; Dako/Agilent)<br>Anti-SSTR2A (UMB-1; Abcam)
Validation	SY38/M0076 (from manufacturer): "Specificity: In Western blot analysis of human neuroblastoma cell lysate, the antibody labels a major band at 40 kDa corresponding to the expected molecular weight of synaptophysin." Reference, IHC: Wiedenmann et al, Cell, 41:1017-1028 (1985)<br>MAB319/PHE5 (from manufacturer): "The antibody has been extensively documented on immunohistochemical studies in deparaffinized methacarn fixed tissue, where it is a sensitive marker of neuroendocrine cells and tumors."<br>H209 (from manufacturer): "In IHC, the antibody labels entero-chromaffine (EC) cells in colon."<br>UMB-1 (from manufacturer): "Suitable for: WB, IHC-P." "The specificity of ab134152 was demonstrated in a Somatostatin Receptor 2-deficient mouse model. Refer to Fischer T et al, J Clin Endocrinol Metab 93:4519-24 (2008)."

Human research participants

Policy information about studies involving human research participants

Population characteristics	Median age at surgery was 76 years, ranging from 48 to 81. 6 (55%) were female and 5 (45%) were male. None had family history of SI-NET or other neuroendocrine tumors. 7/11 (64%) underwent radical surgery. The median WHO 2010 grade was 1, ranging between 1 and 2.
Recruitment	The overall eligible patient cohort consisted of patients with multifocal SI-NET cared for at Sahlgrenska Univerisity Hospital. Sahlgrenska University Hospital is a regional center with a primary responsibility for work-up, treatment and follow up of SI-NET patients within a region with approximately 1.5 million inhabitants. Patients included in the study cohort consists of of a subset of patients that 1) agreed to participate in the study, 2) were treated with surgical resection at Sahlgrenska University Hospital and 3) underwent resection of multiple intestinal SI-NET and lymphnode/liver metasases. Participant enrollment has been close to 100% for several years, and is thus unlikely to bias the results. The minority of patients that were not offered surgical treatment and therefore could not be included in the eligible participant cohort represent a possible selection bias. The choice to refrain from surgical treatment is only based on surgical risk with regards to co-morbidities and it seems unlikely that this represents can affect the results.
Ethics oversight	Regionala etikprövningsnämden i Göteborg / Central ethical review board Gothenburg. All patients referred to Sahlgrenska University Hospital with a confirmed preoperative diagnosis of SI-NET are given verbal and written information regarding current research projects by a research nurse. In-person consent is obtained. In accordance with previous and current ethics permits, prior to 2018, consent was obtained verbally and directly documented in the patients medical journal, after 2018 a separate written consent is obtained and securely stored within hospital premises.

Note that full information on the approval of the study protocol must also be provided in the manuscript.
